# Addressing social determinants of noncommunicable diseases in primary care: a systematic review

**DOI:** 10.2471/BLT.19.248278

**Published:** 2020-05-01

**Authors:** Luke N Allen, Robert W Smith, Fiona Simmons-Jones, Nia Roberts, Rory Honney, Jonny Currie

**Affiliations:** a Nuffield Department of Primary Care Health Sciences, University of Oxford, Radcliffe Primary Care Building, Radcliffe Observatory Quarter, Woodstock Rd, Oxford OX2 6GG, England.; b Nuffield Department of Population Health, University of Oxford, Oxford, England.; c Essex County Council, Essex, England.; d Bodleian Health Care Libraries, University of Oxford, Oxford, England.; e Primary Care and Population Sciences Unit, University of Southampton, Southampton, England.; f Public Health Wales, Cardiff, Wales.

## Abstract

**Objective:**

To explore how primary care organizations assess and subsequently act upon the social determinants of noncommunicable diseases in their local populations.

**Methods:**

For this systematic review we searched the online databases of PubMed®, MEDLINE®, Embase® and the Health Management Information Consortium from inception to 28 June 2019, along with hand-searching of references. Studies of any design that examined a primary care organization assessing social determinants of noncommunicable diseases were included. For quality assessment we used Cochrane’s tool for assessing risk of bias in non-randomized studies of interventions. We used narrative data synthesis to appraise the extent to which the assessments gathered data on the domains of the World Health Organization social determinants of health framework.

**Findings:**

We identified 666 studies of which 17 were included in the review. All studies used descriptive study designs. Clinic-based and household surveys and interviews were more commonly used to assess local social determinants than population-level data. We found no examples of organizations that assessed sociopolitical drivers of noncommunicable diseases; all focused on sociodemographic factors or circumstances of daily living. Nevertheless, the resulting actions to address social determinants ranged from individual-level interventions to population-wide measures and introducing representation of primary care organizations on system-level policy and planning committees.

**Conclusion:**

Our findings may help policy-makers to consider suitable approaches for assessing and addressing social determinants of health in their domestic context. More rigorous observational and experimental evidence is needed to ascertain whether measuring social determinants leads to interventions which mitigate unmet social needs and reduce health disparities.

## Résumé

**Objectif:**

Étudier la façon dont les organismes de soins primaires évaluent et ensuite agissent sur les déterminants sociaux des maladies non transmissibles au sein de leur population locale.

**Méthodes:**

Pour cette revue systématique, nous avons mené nos recherches dans les bases de données en ligne de PubMed®, MEDLINE®, Embase® ainsi que du Health Management Information Consortium, depuis sa création jusqu'au 28 juin 2019, mais aussi cherché manuellement plusieurs références. Quel que soit leur modèle, toutes les études qui portaient sur des organismes de soins primaires évaluant les déterminants sociaux des maladies non transmissibles ont été incluses. Afin de mesurer la qualité, nous avons employé l'outil Cochrane destiné à déterminer le risque de biais dans les études non randomisées sur les interventions. Nous avons également établi une synthèse narrative de données, pour définir dans quelle mesure les évaluations récoltaient des données dans les domaines couverts par les déterminants sociaux de la santé caractérisés par l'Organisation mondiale de la Santé.

**Résultats:**

Nous avons identifié 666 études, dont 17 figurent dans cette revue. Toutes avaient eu recours à des modèles descriptifs. Les enquêtes et entretiens réalisés dans les cliniques et auprès des ménages étaient plus fréquemment utilisés que les données sur l'ensemble de la population pour repérer les déterminants sociaux à l'échelle locale. Nous n'avons trouvé aucun exemple d'organisme tenant compte des moteurs sociopolitiques en lien avec les maladies non transmissibles; tous se concentraient sur des facteurs sociodémographiques ou des conditions de vie spécifiques. Néanmoins, les actions visant à avoir un impact sur les déterminants sociaux étaient multiples: des interventions au niveau individuel jusqu'aux mesures applicables à l'intégralité de la population, en passant par une représentation inédite des organismes de soins primaires dans les stratégies de système et les comités de planification.

**Conclusion:**

Nos découvertes pourraient aider les législateurs à envisager des approches plus adaptées pour évaluer et aborder les déterminants sociaux de la santé dans leur contexte national. Des observations et preuves expérimentales supplémentaires sont nécessaires, afin de vérifier si le fait d'identifier les déterminants sociaux engendre des actions concrètes qui répondent partiellement aux besoins sociaux non satisfaits et réduit les inégalités sanitaires.

## Resumen

**Objetivo:**

Estudiar cómo las organizaciones de atención primaria evalúan y aplican posteriormente los determinantes sociales de las enfermedades no transmisibles en sus poblaciones locales.

**Métodos:**

Para esta revisión sistemática se realizaron búsquedas en las bases de datos en línea de PubMed®, MEDLINE®, Embase® y el Health Management Information Consortium desde su inicio hasta el 28 de junio de 2019, junto con una búsqueda manual de referencias. Se incluyeron estudios de todos los diseños que analizaron a una organización de atención primaria en la que se evaluaban los determinantes sociales de las enfermedades no transmisibles. Para la evaluación de la calidad se utilizó la herramienta de Cochrane para evaluar el riesgo de sesgo en los estudios no aleatorizados de las intervenciones. Se utilizó la síntesis narrativa de datos para evaluar el alcance de las evaluaciones para recopilar datos sobre los dominios del marco de los determinantes sociales de la salud de la Organización Mundial de la Salud.

**Resultados:**

Se identificaron 666 estudios, de los cuales 17 se incluyeron en la revisión. Todos los estudios utilizaron diseños de estudio descriptivos. Se utilizaron las encuestas y las entrevistas en los consultorios y en los hogares con mayor frecuencia que los datos a nivel de la población para evaluar los determinantes sociales locales. No se encontraron ejemplos de organizaciones que evaluaran los factores sociopolíticos determinantes de las enfermedades no transmisibles, ya que todas se centraban en los factores sociodemográficos o en las circunstancias de la vida cotidiana. No obstante, las medidas que se adoptaron para abordar los determinantes sociales fueron desde intervenciones a nivel individual hasta medidas a nivel de la población y la integración de la representación de las organizaciones de atención primaria en los comités de planificación y de políticas a nivel del sistema.

**Conclusión:**

Los resultados obtenidos pueden ayudar a los responsables de formular las políticas a considerar los enfoques adecuados para evaluar y abordar los determinantes sociales de la salud en su contexto nacional. Se necesitan evidencias observacionales y experimentales más rigurosas para determinar si la medición de los determinantes sociales conduce a las intervenciones que mitigan las necesidades sociales que no se atienden y que reducen las desigualdades en la salud.

## ملخص

الغرض اكتشاف كيفية قيام مؤسسات الرعاية الأولية بتقييم المحددات الاجتماعية للأمراض غير المعدية في مجتمعات السكان المحلية، والعمل بناء على أساس ذلك.

الطريقة بالنسبة لهذه المراجعة المنهجية، فقد قمنا بالبحث في قواعد البيانات الخاصة بكل من ®PubMed و®MEDLINE و®Embase عبر الإنترنت، وكونسورتيوم معلومات الإدارة الصحية منذ إطلاقها وحتى 28 يونيو/حزيران 2019، جنبًا إلى جنب مع البحث اليدوي في المراجع. كذلك تم تضمين الدراسات الخاصة بأي تصميم قام بفحص أية منظمة للرعاية الأولية تقوم بتقييم المحددات الاجتماعية للأمراض غير المعدية. لتقييم الجودة، قم باستخدام أداة Cochrane لتقييم خطر التحيز في دراسات التدخلات غير العشوائية. لقد استخدمنا أسلوب تجميع البيانات السردية، لتقييم المدى الذي تم بناء عليه جمع بيانات التقييمات، حول مجالات المحددات الاجتماعية لإطار العمل الصحي التابع لمنظمة الصحة العالمية.

النتائج قمنا بتحديد 666 دراسة، مع تضمين 17 منها في المراجعة. استخدمت جميع الدراسات تصاميم دراسة وصفية. كانت المسوحات والمقابلات الشخصية في العيادات والمنازل أكثر استخداماً بشكل شائع لتقييم المحددات الاجتماعية المحلية، عن البيانات على مستوى السكان. لم نعثر على أمثلة للمؤسسات التي قامت بتقييم الدوافع الاجتماعية والسياسية للأمراض غير المعدية؛ فقد قامت جميعها بالتركيز على الدوافع الاجتماعية والسكانية أو ظروف الحياة اليومية. وعلى الرغم من ذلك، فإن الإجراءات الناتجة للتعامل مع المحددات الاجتماعية قد تراوحت من التدخلات على المستوى الفردي إلى التدابير على مستوى السكان، مع إدخال تمثيل لمؤسسات الرعاية الأولية في لجان السياسات والتخطيط على مستوى النظام.

الاستنتاج إن النتائج التي توصلنا إليها، قد تساعد واضعي السياسات في التفكير بشأن أساليب مناسبة لتقييم والتعامل مع المحددات الاجتماعية للصحة في سياقها المحلي. هناك حاجة إلى أدلة تجريبية وملاحظة جادة للتأكد مما إذا كان قياس المحددات الاجتماعية قد يؤدي إلى تدخلات تخفف من الاحتياجات الاجتماعية غير المستوفاة، وتقلل الفوارق الصحية. ****

## 摘要

**目的:**

探讨初级护理机构如何评估其当地人口非传染性疾病的社会决定因素并采取相应应对措施。

**方法:**

此系统评审中，我们在线搜索了 PubMed®、MEDLINE®、Embase® 和卫生管理信息数据库 (HMIC) 自创立起至 2019 年 6 月 28 日的数据，并手动搜索了相关参考文献。本研究纳入了对评估非传染性疾病社会决定因素的初级护理机构的全部设计的研究。对于质量评估，我们利用 Cochrane 的工具评估非随机性干预研究中的偏差风险。我们使用叙述性数据汇总方法评估此次评估在世界卫生组织卫生框架社会决定因素领域内收集数据的范围。

**结果:**

我们确定了 666 项研究，其中 17 项纳入此次评审中。全部研究均采用描述性研究设计。基于临床和家庭的调查和访谈比人口水平的数据更常用来评估当地的社会决定因素。我们没有发现有关组织评估非传染性疾病的社会政治驱动因素的事例，所有研究均关注于社会人口特征因素或日常生活环境因素。然而，解决社会决定因素的相应措施包括从个人层面干预到人口范围的措施，以及介绍初级护理组织在系统层面政策和规划委员会方面的代表。

**结论:**

我们的发现可能会有助于政策制定者考虑合适的方法来评估和解决其本国环境中的卫生社会决定因素。需要更多有力的观察和实验证据，来确定测量社会决定因素是否会导致相关干预措施，来减轻未得到满足的社会需求并缩小卫生差距。

## Резюме

**Цель:**

Изучить, как учреждения, оказывающие первичную медико-санитарную помощь, оценивают социальные детерминанты неинфекционных заболеваний в местной популяции и действуют на их основании.

**Методы:**

С целью систематического обзора авторы провели поиск данных в доступных в режиме реального времени базах данных PubMed®, MEDLINE®, Embase® и информационного консорциума управления здравоохранения в период с момента создания баз до 28 июня 2019 года с одновременным поиском ссылок вручную. Рассматривались любые исследования, в которых изучалась оценка социальных детерминант неинфекционных заболеваний учреждениями, оказывающими первичную медико-санитарную помощь. Для оценки качества использовался метод Кокрана, оценивающий риск необъективности в нерандомизированных исследованиях разного рода вмешательств. Авторы использовали синтез данных описательного характера для оценки объема сбора данных проводимыми исследованиями по социальным детерминантам систем здравоохранения на разных доменах Всемирной организации здравоохранения.

**Результаты:**

Было обнаружено 666 исследований, 17 из которых были включены в обзор. Все исследования носили дескриптивный характер. Для оценки местных социальных детерминант чаще использовались опросы в клинике и анкетирование и опросы семей, чем данные по населению в целом. Авторам не удалось найти примеры организаций, которые бы оценивали социально-политические причины распространения неинфекционных заболеваний. Все исследования касались социально-демографических факторов или обстоятельств повседневной жизни. Тем не менее соответствующие мероприятия по результатам оценки социальных детерминант варьировались от индивидуальных вмешательств до общенациональных мер, а также была обеспечена репрезентативность учреждений, оказывающих первичную медико-санитарную помощь, в политике на уровне системы и в комитетах планирования.

**Вывод:**

Результаты исследования могут помочь лицам, ответственным за принятие стратегических решений, рассмотреть приемлемые подходы к оценке социальных детерминант здоровья в контексте соответствующих стран и решению связанных с ними проблем. Необходимы более строгие наблюдения и экспериментальные данные, чтобы определить, приводит ли измерение социальных детерминант к вмешательствам, смягчающим последствия неудовлетворенных социальных потребностей и уменьшающим неравенство в области здравоохранения.

## Introduction

Primary health care is a whole-of-society approach to health that depends on integrated primary care and essential public health functions; empowered people and communities; and multisectoral policy and action.^1^ World Health Organization (WHO) Member States have unanimously committed to use primary health care as the main vehicle for attaining universal health coverage.^1^^–^^4^

Given that health services are thought to be responsible for only a fifth of health outcomes,^5^^,^^6^ primary care systems are increasingly being reoriented to proactively assess and address local social determinants of health,^7^^–^^12^ particularly the social determinants of noncommunicable diseases. These diseases are responsible for over 70% of global mortality (41 million out of 58 million annual deaths).^13^^,^^14^ Socioeconomic factors are associated with exposure to behavioural risk factors for and mortality from noncommunicable diseases,^15^^,^^16^ and exposure to noncommunicable disease risk factors, such as poverty, tobacco or unhealthy foods, occurs at the local levels where people live and work. Primary care organizations therefore have a strategic role to play in prevention and control of noncommunicable diseases. This role was emphasized in WHO’s Commission on the Social Determinants of Health 2007 report, *Challenging inequity through health systems*.^17^ Reforming primary care to engage with public health functions in collaboration with community stakeholders is also a way of enacting the commitments made in the Declaration of Astana on revitalizing primary health care in the 21st century.^12^

Social determinants, which have been defined as “the conditions in which people are born, grow, work, live and age, and the wider set of forces and systems shaping the conditions of daily life,”^18^ account for approximately half of all variation in health outcomes.^6^^,^^19^^–^^22^ The WHO Commission on the Social Determinants of Health urged Member States to “go beyond contemporary concentration on the immediate causes of disease” to focus on these “causes of the causes.”^17^ The Commission’s conceptual framework has three elements covering different domains.^23^ First are the sociopolitical factors that influence distributions of health outcomes across populations (such as social and economic policy, cultural norms and societal values). These factors can be distinguished from the sociodemographic factors according to which health is unequally distributed (such as income, education, gender and ethnicity or race) and the circumstances of daily life which more directly influence people’s exposure and vulnerability to adverse health outcomes (such as age, housing and food security, sanitation, health behaviours and access to health care). The Commission’s framework for action on the social determinants of health conceptually differentiates policy interventions in terms of their target population: individuals (microlevel), communities (mesolevel) and whole of society (macrolevel).^23^

Countries as diverse as Azerbaijan, Ethiopia and the United Kingdom of Great Britain and Northern Ireland are currently reforming their primary care systems to address the social determinants of noncommunicable diseases. Yet it remains unclear how primary care organizations can most effectively collect person- and population-level data to subsequently act upon identified needs.^7^^,^^12^^,^^24^ Much of the existing research on assessing social determinants of health has focused on secondary care settings in high-income countries.^24^^–^^27^

To address this gap, we systematically reviewed the literature to collate examples of primary care organizations that had performed assessments of the social determinants of noncommunicable diseases with the intention of subsequently acting upon the knowledge generated. We aimed to determine which social determinants of health are most commonly assessed, the approaches used to collect data, what actions resulted and what barriers and enablers were reported. A secondary aim was to examine whether routine assessments of the social determinants of health were more likely to report actions than were non-routine, or “one-off” assessments.

## Methods

Our systematic review followed Cochrane guidance^28^ and was reported in accordance with the 2009 Preferred Reporting Items for Systematic Reviews and Meta-Analyses (PRISMA) statement.^29^ The protocol was registered with the PROSPERO prospective register of systematic reviews in July 2019 (CRD42019141291).

### Search strategy

We searched the online databases of MEDLINE^®^, Embase^®^, PubMed^®^ and the Health Management Information Consortium on 28 June 2019 without restrictions on language, period or country (the full search strategy is available in the data repository).^30^ We also manually searched the reference lists of included studies and contacted key authors and policy experts at WHO to find any additional studies.

We included all study designs that examined one or more historic or contemporary primary care organization committing resources to assessing the social determinants of noncommunicable diseases in their local community with the intention of subsequently intervening. Studies that described the assessment activities of specific primary care staff cadres, such as community health workers, were also included.

We excluded papers such as editorials and reviews that did not present primary data, but we hand-searched their reference lists and included any eligible original studies. As our focus was real-world practice, we excluded papers that only described theoretical models or unrealized organizational plans. We excluded papers that described single-issue initiatives for narrow subpopulations if these were not based on community-wide assessments. We excluded studies that focused exclusively on paediatric populations, unless the primary care organization was a community-based paediatric service, so that their entire patient population was included. We also excluded studies that did not report the intention of assessing impact on services or health outcomes.

Two researchers independently screened all titles and abstracts and then the full texts. Cohen’s kappa coefficient (*κ*) and percentage agreement were calculated for both stages of screening.^31^ Disagreement was resolved by discussion and arbitration by a third researcher if necessary.

### Data extraction, synthesis and analysis

Two researchers independently extracted the data using a form developed from the Cochrane template,^32^ including study design, setting, assessment approach, factors identified by the assessment and subsequent community-level actions. We used narrative data synthesis to appraise whether the health assessments in the included studies gathered data on the domains of the WHO social determinants of health framework.^23^

We used also used Geoffrey Roses’s population versus high risk conceptual approach to assess whether different organizations assessed and addressed social determinants of health at the individual and/or community levels.^33^ Given the lack of formal consensus around the precise boundaries of the social determinants of health,^34^^,^^35^ we included all data that the authors self-identified as social determinants, including individual-level sociodemographic characteristics.

### Risk of bias assessment

Two reviewers independently assessed the risk of bias of each included study using Cochrane’s risk of bias tool for non-randomized studies of interventions.^36^ Guided by the Cochrane handbook,^28^ we rated studies as having low, moderate, serious or critical risk of bias across seven domains and overall.

## Results

### Search results

**Fig. 1:**
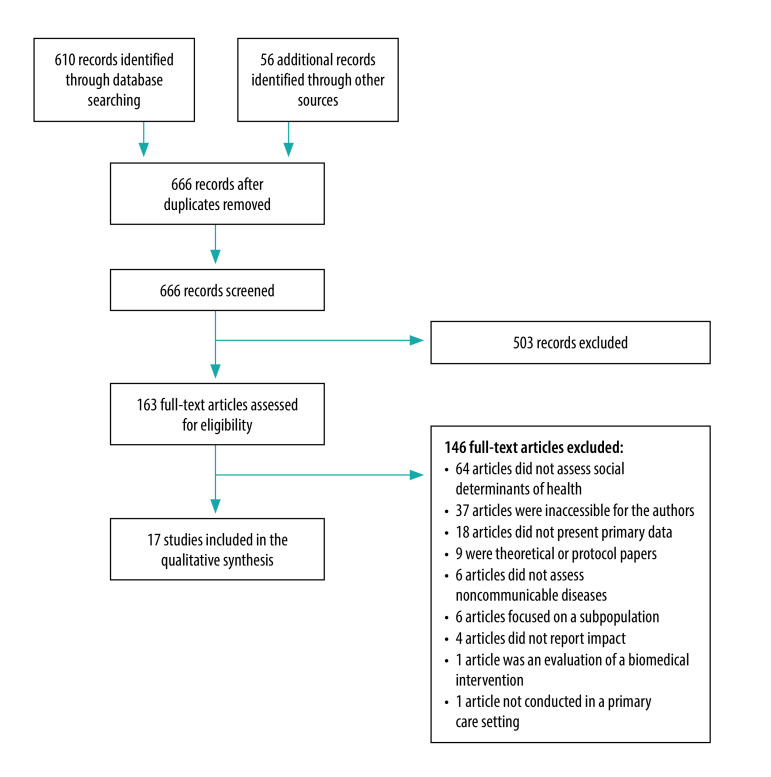
Flow diagram of selection of papers for inclusion in the review of approaches to addressing social determinants of health in primary care

Our searches identified 666 records of which 17 studies from 15 different primary care organizations met the inclusion criteria after two stages of screening (Fig. 1). Cohen’s *κ* was > 0.80 and agreement was > 95% at every stage of screening.

### Study characteristics

The characteristics of the included studies are summarized in Table 1. All studies took place in high- or middle-income countries, with nine studies conducted in the United States of America (USA),^37^^–^^41^^,^^43^^,^^47^^,^^48^^,^^50^ three in South Africa,^42^^,^^46^^,^^49^ three in Canada^51^^–^^53^ and two in the United Kingdom (one in England^45^ and one in Wales^44^).

**Table 1:** Characteristics of studies included the systematic review of approaches to assessing and addressing social determinants of health in primary care

Study	City or region, country	Study type	Primary care organization	Population served
Institute of Medicine, 1984^37^	Checkerboard area of the Navajo Nation, New Mexico, USA	Case study	System of satellite primary health-care clinics	14 000 patients from largely indigenous communities
Institute of Medicine, 1984^38^	Bailey, Colorado, USA	Case study	Fee-for service rural family medicine centre with 2 physicians and 5 nursing staff	7 280 patients. Low representation of adult patients over 65 years of age compared with the broader community
Institute of Medicine, 1984^39^	East Boston, Massachusetts, USA	Case study	1 large, interprofessional, fee-for-service, group health-care practice	Approximately 32 000 residents of a socioeconomically deprived region of inner-city Boston
Institute of Medicine,1984^40^	The Bronx, New York, USA	Case study	1 publicly funded, interprofessional, community health centre	20 000 patients residing in 9 urban catchment areas of an area of inner-city New York
Institute of Medicine,1984^41^	Edgecombe County, North Carolina, USA	Case study	1 multidisciplinary, private fee-for-service, primary health-care practice	Rural community of approximately 12 000 residents
Tollman,1994^42^	Pholela District, KwaZulu-Natal, South Africa	Case study	1 interprofessional, publicly funded, rural primary health-care centre	Approximately 10 000 patients in the 1940s
Williams & Jaén, 2000^43^	Cleveland, Ohio, and Buffalo, New York, USA	Case study	11 predominantly small to medium-sized primary health-care group practices	8 urban and largely marginalized communities, 1 suburban and 1 semi-rural community
Fone et al., 2002^44^	Caerphilly County Borough, Wales, United Kingdom	Cross-sectional study	Local authorities and local health groups	Approximately 170 120 residents of socioeconomically diverse communities within the Gwent health authority, south-east Wales
Horne and Costello, 2003^45^	Hyndburn, England, United Kingdom	Rapid participatory appraisal study	5 publicly funded primary health-care teams	1 district in north-west England
Bam et al., 2013^46^	Tshwane District, Gauteng South Africa	Case study	9 primary care health posts	2 000 to 3 000 households in the most socioeconomically deprived sub-districts of Tshwane District
Hardt et al., 2013^47^	Alachua County, Florida, USA	Case study	Academic health system with primary health-care practices	Urban community of approximately 124 354 residents with large student population
Gottlieb et al. 2015^48^	Baltimore, Maryland, USA	Case study	Urban teaching hospital paediatric clinic	Families attending Johns Hopkins Children’s Center Harriet Lane clinic
Jinabhai et al., 2015^49^	Eastern Cape, Free State, Mpumalanga, Limpopo, Gauteng, Northern Cape, North West, South Africa	Rapid participatory appraisal study	Interprofessional ward-based outreach teams constituting primary health and social care providers	Over 673 000 households across 7 provinces
Page-Reeves et al., 2016^50^	Albuquerque, New Mexico, USA	Mixed-methods pilot study	2 academic family medicine clinics and 1 community health centre	Large, low-income patient populations
Pinto et al., 2016^51^	Toronto, Ontario, Canada	Case study	5 interprofessional academic primary health-care clinics	Sociodemographically diverse inner-city patient population of approximately 35 000 patients
Lofters et al., 2017^52^	Toronto, Ontario, Canada	Retrospective cohort study	6 interprofessional, publicly funded, academic primary health-care clinics	Sociodemographically diverse inner-city population of approximately 45 000 patients. Study sample focused on adults eligible for publicly funded colorectal, cervical or breast cancer screening programmes
Pinto & Bloch, 2017^53^	Toronto, Ontario, Canada	Case study	6 interprofessional, publicly funded, academic primary health-care clinics	Sociodemographically diverse inner-city population of approximately 45 000 patients

Six of the 17 records were published after 2014 while the oldest records came from the second volume of the 1984 Institute of Medicine report on community-oriented primary care. This volume described case studies of USA primary care organizations, five of which met our inclusion criteria.^37^^–^^41^ One record presented findings from a community-oriented primary care project in South Africa in the 1940s.^42^ Among the remaining papers, a further seven were descriptive case studies,^42^^,^^43^^,^^46^^–^^48^^,^^51^^,^^53^ making this the most prevalent study design. There was one retrospective cohort study,^52^ two rapid participatory appraisals,^45^^,^^49^ a mixed-methods pilot study^50^ and a cross-sectional study that also provided a narrative account of efforts to address social determinants.^44^ Three papers reported on the same Canadian primary care organization.^51^^–^^53^

Ten of the 17 studies described efforts led by primary health-care clinicians who had been actively involved in new initiatives to gather data on the social determinants of health in their local communities.^43^^–^^48^^,^^50^^–^^53^ Most papers aimed to describe novel assessment initiatives. For three papers these initiatives were nested within evaluations of broader interventions.^37^^–^^41^^,^^46^^,^^49^

### Assessment activities reported

The included studies examined a wide range of domains of the social determinants of health, 20 of which were captured by two or more studies (Table 2; available at: http://www.who.int/bulletin/volumes/98/11/19-248278). Circumstances of daily life and indicators of socioeconomic position were the most commonly assessed. No studies assessed social cohesion. Assessed in 11 studies (9 organizations), the most common indicators of socioeconomic position were measures of income or financial situation. Additional population stratification factors, such as race and ethnicity, nationality, religion, disability, sexual orientation and language were only assessed in two recent studies. Only two studies (reporting on the same primary care organization) explicitly sought to assess both sex and gender identity.^51^^,^^53^ Two studies did not report which specific social determinants of health data they were assessing.^46^^,^^49^ No studies assessed any of the wider sociopolitical factors.

**Table 2:** Sociodemographic data collected by each reviewed study in the systematic review of approaches to assessing and addressing social determinants of health in primary care

Domains of WHO framework assessed	Institute of Medicine, 1984^37^	Institute of Medicine, 1984^37^	Institute of Medicine, 1984^37^	Institute of Medicine, 1984^37^	Institute of Medicine, 1984^37^	Tollman, 1994^42^	Williams & Jaén, 2000^43^	Fone et al., 2002^44^	Horne & Costello, 2003^45^	Bam et al., 2013^46^	Hardt et al., 2013^47^	Gottlieb et al., 2015^48^	Jinabhai et al., 2015^49^	Page-Reeves et al., 2016^50^	Pinto et al., 2016^51^	Lofters et al., 2017^52^	Pinto & Bloch, 2017^53^
**Circumstances of daily life**																	
Material circumstances																	
Food security and diet	No	No	No	No	No	Yes	Yes	No	No	No	No	Yes	No	Yes	No	No	No
Housing	Yes	Yes	No	No	No	Yes	No	Yes	Yes	No	No	Yes	No	Yes	Yes	Yes	Yes
Transport	No	No	Yes	No	No	No	Yes	No	No	No	No	No	No	Yes	No	No	No
Health and social care access	Yes	Yes	No	No	No	Yes	Yes	Yes	Yes	No	Yes	Yes	No	No	No	No	No
Safety and crime	No	No	Yes	No	No	No	Yes	No	Yes	No	Yes	No	No	Yes	No	No	No
Water and sanitation	No	No	No	No	No	Yes	No	No	No	No	No	No	No	No	No	No	No
Childcare access	No	No	No	No	No	No	No	No	No	No	No	Yes	No	Yes	No	No	No
Health insurance	No	No	No	Yes	No	No	No	No	No	No	Yes	Yes	No	No	No	No	No
Social cohesion	No	No	No	No	No	No	No	No	No	No	No	No	No	No	No	No	No
Psychosocial factors																	
Social relationships and support	Yes	No	Yes	Yes	No	Yes	No	No	Yes	No	Yes	No	No	Yes	No	No	No
Behaviours																	
Substance use	Yes	No	No	No	Yes	No	No	No	No	No	No	No	No	Yes	No	No	No
Biological factors																	
Age	Yes	Yes	No	No	No	Yes	No	No	No	No	Yes	No	No	No	Yes	Yes	Yes
Sex	Yes	Yes	No	No	No	Yes	No	No	No	No	No	No	No	No	Yes	Yes	Yes
**Population stratifiers and indicators of socioeconomic position**																	
Education	No	No	No	No	Yes	Yes	No	Yes	No	No	Yes	Yes	No	Yes	No	No	No
Occupation and employment	No	No	Yes	No	Yes	Yes	No	Yes	Yes	No	No	Yes	No	Yes	No	No	No
Income and finances	No	No	Yes	Yes	No	No	Yes	Yes	Yes	No	Yes	Yes	No	Yes	Yes	Yes	Yes
Gender	No	No	No	N0	No	No	No	No	No	No	No	No	No	No	Yes	No	Yes
Race and ethnicity	No	No	No	No	No	No	No	No	No	No	No	No	No	No	Yes	Yes	Yes
Other: not specified in WHO framework																	
Nationality	No	No	No	No	No	No	No	No	No	No	No	No	No	No	Yes	Yes	Yes
Religion	No	No	No	No	No	No	No	No	No	No	No	No	No	No	Yes	Yes	Yes
Disability	No	No	No	No	No	No	No	No	No	No	No	No	No	No	Yes	Yes	Yes
Sexual orientation	No	No	No	No	No	No	No	No	No	No	No	No	No	No	Yes	Yes	Yes
Language	No	No	No	No	No	No	No	No	No	No	No	No	No	No	Yes	Yes	Yes
**Wider sociopolitical factors**																	
Governance	No	No	No	No	No	No	No	No	No	No	No	No	No	No	No	No	No
Policy	No	No	No	No	No	No	No	No	No	No	No	No	No	No	No	No	No
Cultural and social norms and values	No	No	No	No	No	No	No	No	No	No	No	No	No	No	No	No	No

WHO: World Health Organization.

Notes: We categorized data according to the WHO’s conceptual framework for action on the social determinants of health.^23^ Two studies did not report on the specific social determinants of health data.^46^^,^^49^ Two studies reported on the same intervention.^51^^,^^53^ Some studies assessed other social determinants of health data: free-text capture of sociodemographic information;^48^ Jarman index of deprivation;^45^ Townsend index of deprivation;^44^ self-reported health;^44^^,^^51^^,^^53^ and perceived community health needs.^43^^,^^45^

### Approach to data collection

Ten studies described routine data collection activities on the social determinants of health,^37^^,^^39^^,^^40^^,^^42^^,^^46^^,^^48^^,^^49^^,^^51^^–^^53^ six described non-routine or one-off assessments,^41^^,^^43^^–^^45^^,^^47^^,^^50^ and one study was unclear.^38^ Almost all organizations that employed routine assessments collected individual-level data from patients or their proxies, often within clinic receptions or waiting areas (Table 3). One study additionally linked individual records to neighbourhood median household income level^52^ and another study supplemented patient-level data with routine household surveys conducted by community health workers.^49^ Greater heterogeneity in data domains and collation methods was observed among studies describing non-routine social determinants of health assessments. Aggregate-level administrative data on social determinants of health were collated for the primary care organization’s catchment areas (such as neighbourhood deprivation) or such data were linked to patient rosters using identifiers such as postcode,^44^^,^^45^^,^^47^ household surveys,^40^^,^^41^^,^^46^ in-clinic surveys^50^ and telephone interviews.^43^

**Table 3:** Source of individual- and population-level data and types of actions by primary care organizations involved in assessing and addressing the social determinants of health

Study	Sources of individual-level data	Sources of population-level data	Types of action
Institute of Medicine, 1984^37^	Unclear	Unclear	Not reported
Institute of Medicine, 1984^38^	Unclear	Unclear	Not reported
Institute of Medicine, 1984^39^	Patient or proxy in a clinic (unspecified setting)	Not collected	New services for specific subgroups New non-health services Introduction of new legislation or policies
Institute of Medicine,1984^40^	Patient or proxy in clinic waiting room. Home visits	Not collected	New services for specific subgroups New non-health services
Institute of Medicine,1984^41^	Household visits	Unclear	Not reported
Tollman,1994^42^	Home visits. Individuals in clinics	Unclear	Individual-focused interventions. New non-clinical services
Williams & Jaén, 2000^43^	Patient or proxy telephone interviews	Not collected	New services for specific subgroups
Fone et al., 2002^44^	Not collected	Administrative data: held by another agency, not publicly available	New clinical services that benefit the entire community New representation in policy and planning processes
Horne and Costello, 2003^45^	Key informant interviews. Focus groups	Administrative data: unclear	New services for specific subgroups New clinical services that benefit the entire community New non-health services New representation in policy and planning processes
Bam et al., 2013^46^	Household visits	Not collected	Not reported
Hardt et al., 2013^47^	Not collected	Publicly available data and non-publicly available held by other agencies	New clinical services that benefit the entire community New integrated health and social services Introduction of new legislation or policies
Gottlieb et al. 2015^48^	Patient or proxy in a clinic (unspecified setting)	Not collected	Individual-focused interventions
Jinabhai et al., 2015^49^	Individuals in clinics. Household visits	Not collected	Individual-focused interventions. New clinical services that benefit the entire community New integrated health and social services. New non-health services New representation in policy and planning processes
Page-Reeves et al., 2016^50^	Patient or proxy in a clinic (unspecified setting)	Not collected	Individual-focused interventions. New clinical services that benefit the entire community Introduction of new legislation or policies
Pinto et al., 2016^51^	Patient or proxy in a clinic waiting room	Not collected	Introduction of new legislation or policies
Lofters et al., 2017^52^	Patient or proxy in a clinic waiting room	Non-publicly available data held by other agencies	Not reported
Pinto & Bloch, 2017^53^	Patient or proxy in a clinic waiting room	Not collected	Not reported

In total, six studies involved the collection of population-level data.^37^^,^^39^^,^^42^^,^^44^^,^^47^^,^^52^ Publicly-available data sources included censuses, state health departments and nongovernmental organizations. Non-publicly accessible sources (those requesting special data requests) included local, regional and federal government departments for health, social services, labour and education, municipal police departments, regional health-planning agencies and certain national data sets.

### Actions reported

**Fig. 2:**
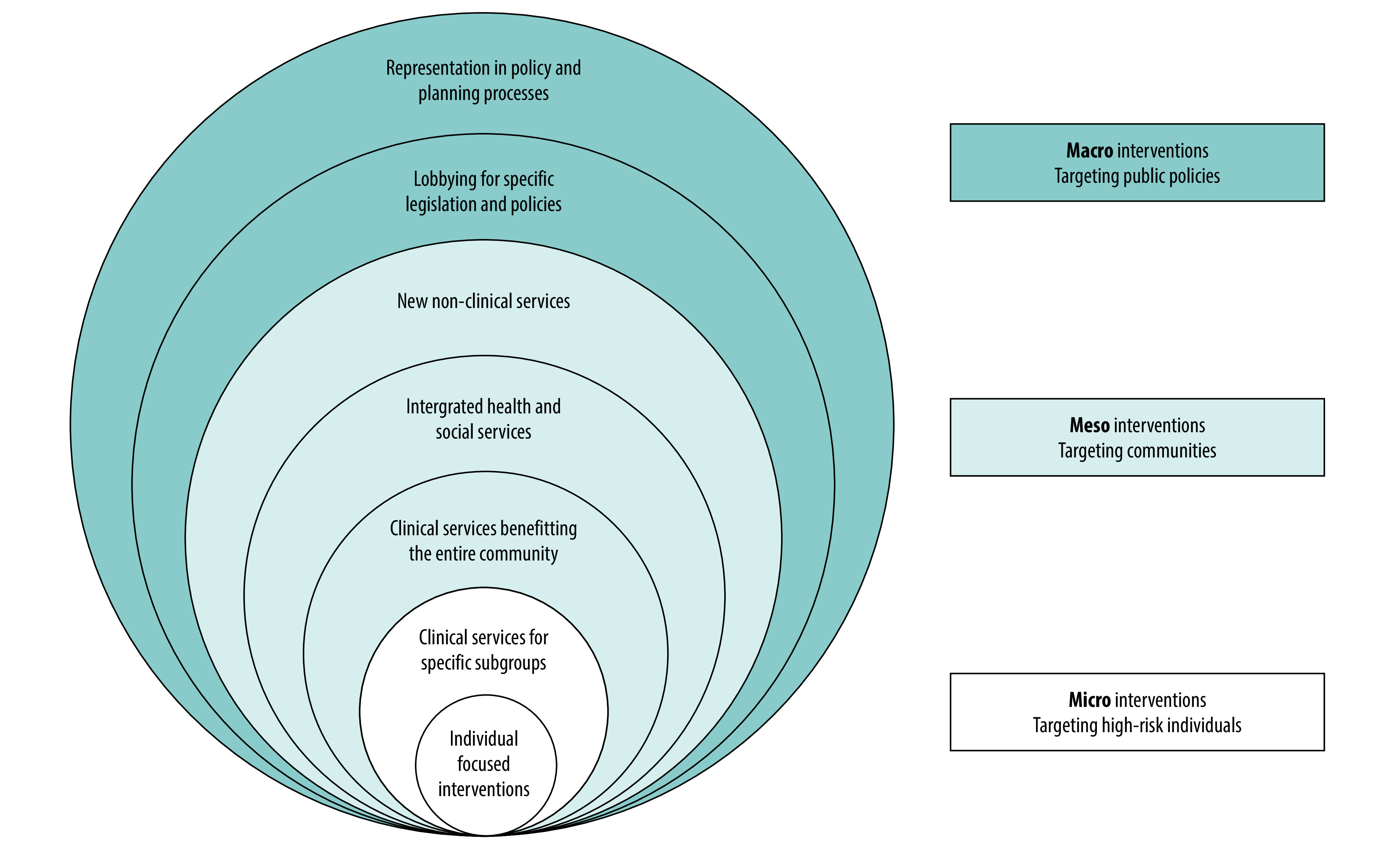
A taxonomy of approaches to translate local data on social determinants of health into action

In addition to action to extend data collection to other primary care sites,^50^^,^^51^ the studies described several other initiatives by primary care organizations to address the social determinants of health (Table 3; Box 1). These initiatives ranged from individual-focused biomedical interventions through to population-level, health-in-all-policy partnerships with local authorities and non-health agencies. Building on the WHO conceptual framework for action on the social determinants of health, we present a new conceptual taxonomy of the different strata addressed by the studied primary care organizations (Fig. 2), dividing actions into macro, meso and micro levels.

Box 1 **Examples of primary care organizations’ actions to address local social determinants of health**

***Microlevel actions: targeting high-risk individuals***

**Individual-focused interventions**

Several study sites identified patients with unmet social needs and provided these individuals with educational materials,^43^ referred them on to relevant services^48^^,^^49^ or connected them with community workers.^42^^,^^50^

**New services for specific subgroups**

Four study sites identified specific subpopulations with high levels of need: people of Asian ancestry,^45^ older adults,^39^^,^^43^ and Cambodian refugees.^40^ New services were created for these groups including: tailored educational materials, health services, and social interventions such as working with landlords to improve rental housing stock;^40^ lobbying for improved transport infrastructure;^39^ and setting up community welfare groups.^45^

***Mesolevel actions: targeting communities***

**New clinical services that benefit the entire community**

Five study sites developed new clinical services that stood to benefit the entire community: relocating pre-existing clinical services and starting a mobile outreach clinic;^47^ launching a health bus and a new practice-based health promotion programme at the local produce market;^45^ extending the scope of clinical services offered by the primary health-care practice;^44^ hiring new community health workers;^50^ and an array of new clinical services offered by ward-based outreach teams.^49^

**New integrated health and social services**

The academic primary health-care practice network in Alachua county, USA, described the creation of a new integrated health and social care community resource centre.^47^ Another study of outreach teams in seven provinces in South Africa described the initiation of multidisciplinary meetings to plan integrated services with local populations.^49^

**New non-clinical services**

After finding that access to local health services was poor, the neighbourhood health centre in Boston in the USA, sought funding for new transport infrastructure. The centre also applied for funding to improve the local housing stock and sought to influence television broadcasting to promote anti-violence messages in response to high homicide rates uncovered by their data.^39^ The Bronx community health centre in New York, USA, worked with landlords to improve housing standards and remove lead-based paint causing respiratory problems identified by their linkage of health and social determinants of health data.^40^ In the study in Hyndburn, England, the primary health-care partnership instigated the establishment of a credit union after finding that debt and low income was a problem for many local residents.^45^ The Pholela project workers in KwaZulu-Natal, South Africa, built new vegetable gardens to help improve the local population’s nutritional status.^42^ South Africa’s ward-based outreach teams in seven provinces also set up several garden projects, as well as helping communities obtain new toilets, water banks, food parcels, child support grants and overarching birth certification.^49^

***Macrolevel actions: targeting public policies***

**Lobbying and introduction of new legislation and policies**

The health-centre partnership in Albuquerque, USA, went beyond delivering new programmes and services to successfully lobby for new legislation (the Community Health Workers Act).^50^ Due in part to a previous initiative,^51^ regional authorities directed all hospitals within central Toronto city, Canada, to begin standardized sociodemographic data collection. The health centre in Boston in the USA modified existing parent counselling protocols to de-emphasize practices that were believed to “condone or may predispose children to violence” in an effort to prevent future violence.^39^ As a result of presenting health disparity hot-spot maps (displaying the location and intensity of socioeconomic issues) in Alachua county, USA, community organizing and advocacy activities were initiated to lobby for better social conditions.^47^

**New representation in policy and planning processes**

Assessing local needs is believed to have strengthened relationships across the primary health-care partnership in Hyndburn, England – primarily between the general practitioner’s surgery and the local government.^45^ The results of the assessment contributed to environmental policy-making and decisions around housing developments and local town regeneration. In South Africa, ward-based outreach teams and their primary care managers established partnerships with local government, nongovernmental organizations, faith organizations, private sector agencies, and local village councils, allowing them to collaboratively develop new services and have a voice in local decision-making.^49^ Finally, as a result of developing a multi-agency data set on health and social inequality in the study in Caerphilly, Wales, representatives from local general medical practices became major partners with local authorities and local communities, contributing to planning processes and participating in development of future community strategies.^44^

We identified several factors as potential facilitators to effective collection, analysis and translation of social determinants of health data into action. At organizational and system levels, facilitating included strong commitment in terms of leadership, funding and human resources, and having health equity embedded within the organization’s strategic plans.^44^^,^^53^ Pre-existing collaborative multisectoral partnerships were also believed to enable multifaceted responses to community health needs identified by social determinant assessments.^47^ Electronic data collection using tablet computers and mobile phones was believed to be feasible and acceptable within both well- and under-resourced clinical settings.^49^^,^^51^ Translating surveys into multiple languages was believed to improve response rates and overall data quality.^51^^–^^53^ Having social determinants of health data entry fields integrated within electronic health records was believed to improve the documentation of unmet social needs and communication of such information across care teams.^48^

Missing data limited the completeness of the primary data collected for more sensitive sociodemographic information such as income. Data representativeness was potentially limited where only a sub-sample of key informants provided social determinants of health information on behalf of the wider community.^43^^,^^51^^,^^52^ Given that population-level data sets were often sourced from multiple organizations, the quality and compatibility of linked data were hindered by variations in data collection time periods, different geographical boundaries and varying coverage of the population.^40^^,^^44^ Linking population-level data to individual-level data was difficult in practices using hard-copy medical records.^39^ The time and human resource investments required for data collection, processing, analysis and reporting was raised as a key challenge for implementing social determinants of health data collection, even in well-resourced primary care settings.^43^^,^^44^^,^^48^^,^^49^ Finally, public trust in the people and organizations collecting social determinants of health data was highlighted as an important consideration for primary data collection.^42^^,^^43^

### Risk of bias assessment

The overall risk of bias was serious for 15 studies and critical for two studies. Due to the reliance on descriptive study designs, most studies provided limited detail regarding who received the social determinants of health assessment intervention and how these interventions were implemented. Another important source of potential bias was in the attribution of actions (that were not pre-specified) to the social determinants of health assessment intervention without comparison to a control group or accounting for other potentially confounding factors. Domain-specific risk of bias assessments are presented in the data repository.^30^

## Discussion

Our review provides primary health-care practitioners and policy-makers with an overview of the approaches taken to assess and address social determinants of noncommunicable diseases by 15 primary care organizations. Although this policy objective is a leading priority for international health systems, we found very few contemporary examples in the peer-reviewed literature that met the inclusion criteria for this study. There was marked heterogeneity in the domains assessed by the different organizations, and no assessments of macro-level sociopolitical factors that influence distributions of health across populations. Organizations tended to collect individual-level data in clinical settings rather than population-level data. There was a broad range of actions targeting individuals, communities and the whole of society. The use of descriptive case studies and an absence of long-term evaluations of health outcomes among the included studies limits the conclusions we can reach about the relative merits of each approach. Nevertheless, the heterogenous approaches described in this paper highlight diverse options for care providers and policy-makers to consider.

Gold-standard approaches to collection of data on the social determinants of health in health-care settings have yet to be identified. Nevertheless, the WHO Commission’s framework^23^ provides a useful starting point for primary care organizations in defining, which specific factors are most relevant to their local context. As observed in our review, the specific domains of the social determinants of health assessed and the methods for data collection will likely vary according to an organization’s purpose for assessment and the resources available to support data collection, analysis and response.

Approximately half of the studies in our review assessed the social determinants of health via individual-level surveys in clinical settings. These data can complement existing biomedical information from medical records. A 2018 scoping review found that a growing number of screening tools are being used around the world to help frontline clinicians collect data on social determinants.^54^ These data are mainly being used in a case-finding capacity to identify individuals with multiple domains of social risk.^55^ The combination of individual-level social data (such as on poverty, housing and food insecurity) and clinical health data (such as blood pressure, cholesterol levels, medications and pre-existing conditions) can help health workers to identify population groups with specific needs and inform the design of appropriate interventions. However, approaches that rely on data collected solely in clinic settings will exclude vulnerable members of the local community who are not registered or do not seek care from primary care providers.^56^^,^^57^ This approach may also fail to capture higher-order and population-wide factors such as welfare policy, transport networks and sanitation.

The other half of the reviewed studies either collected new population-level data or collated pre-existing population-level sociodemographic data sets. These data collection activities were often stand-alone endeavours as opposed to routine, systematized activities. Aggregated community-level data can conceal within-population inequalities, but otherwise tend to provide a more representative picture than extrapolating from patient registries. Whether data are at the individual or population level, it is paramount that primary care organizations carefully consider the representativeness of their data sources.^58^^,^^59^

The actions of the primary care teams in South Africa^49^ and the United Kingdom^44^^,^^45^ model the primary health-care philosophy of integrating public health and primary care functions to engage in intersectoral action.^60^ Nevertheless, we did not find any examples of primary health-care organizations that employed routine systems for collating population-level data on the social determinants of health and that worked collaboratively on an everyday basis. Intersectoral collaboration was highlighted as an enabler of population-level social determinants of health assessment. Yet the reviewed studies suggest that there are challenges to finding the human, financial and technological resources required to build and maintain routine population-level assessment systems on the social determinants of health. These roles can be provided by organizations responsible for planning and resourcing local health and social services (such as health ministries), academic institutions, or those representing primary care professionals (such as professional associations and governing bodies). Contemporary examples include population health data parsed for primary care organizations by Public Health England,^61^ the American Board of Family Medicine and University of Missouri,^62^ and the Slovenian National Institute of Public Health.^63^ There are numerous other systems containing population-level data on a wide range of social determinants of health indicators that are not currently linked to specific primary care practice populations.^64^ Further research should explore how to support the use of these data in primary care to plan local population services that are responsive to community needs.

Assessing the domain of wider sociopolitical factors was another identified gap in data collection by primary care organizations. This omission may be justified given that the purpose of collecting data on the social determinants of health tended to focus on the local community. However, the gap may also stem from a lack of clarity on which measures within this domain are relevant, feasibly measured and actionable for primary care organizations. Understanding how to collect data on wider sociopolitical factors represents an important area for future research. Further gaps include information around funding mechanisms, workforce arrangements and the interface with public health agencies. 

Almost all the primary care organizations used new knowledge on local social determinants of health to design and deliver novel interventions with the goal of reducing health inequalities and improving population health outcomes. These ranged from downstream individual-focused activities like producing educational materials, to upstream health-in-all-policies approaches, such as joining local authority planning and commissioning boards. Similar themes of action are reflected in the 2019 consensus report on the integration of health and social care in the USA.^24^ Our social determinants of health taxonomy of actions builds on previous research^22^ and the WHO Commission’s report^65^ to provide a way of thinking through the various levels where primary care organizations can act to make positive changes.

An important consideration for future research is how, when and where primary care organizations should engage with traditional public health activities. Our review has illustrated the heterogeneity in primary care activities on addressing the social determinants of health. However, despite decades of work to define the characteristics of primary care,^66^ there is no consensus in the health policy community around exactly which activities and functions primary care ought to perform. One approach is unlikely to be suitable for all settings, as different primary care systems have different skills, resources, and cultural expectations. There are already marked contrasts between even closely related systems. While Dutch family physicians recently rejected mooted new public health responsibilities ^67^ almost all general practitioner surgeries in England^68^ have taken on responsibilities for addressing neighbourhood inequalities and improving population health.^69^

Our review has several strengths. It was conducted in line with Cochrane and PRISMA guidelines and used a robust search strategy with independent dual review with good agreement at every stage. The review addresses an important evidence gap for global primary health care and provides detailed and pragmatic insights for clinicians and policy-makers. Limitations of this review primarily relate to the types of studies included. Most were case studies detailing implementation approaches rather than quantifying associations between social determinants of health assessment and subsequent actions and outcomes. In particular, the absence of health outcomes data reported in the included studies highlights the challenges primary care organizations face with finding the resources to evaluate downstream outcomes of interventions that may take several years to manifest. This aligns with public health research more broadly^70^^,^^71^ and social determinants of health-oriented primary care research specifically, where the field is hampered by a lack of randomized controlled trials and of methodological tools for evaluating complex interventions.^72^ To examine whether assessment of the social determinants of health leads to interventions mitigating unmet social needs and health disparities, future research should endeavour to use longer follow-up periods and more rigorous observational and experimental methods.^73^^,^^74^ Despite stating an intention to use social determinants of health data collection to inform action, six of the 17 studies did not report on any actions. We did not find any studies from low-income countries that met the inclusion criteria and our review may also have missed unpublished international examples, thus limiting the generalizability of our findings. Despite these limitations, case studies arguably provide useful evidence for how and why a particular approach was employed.

## Conclusions

Tasked with the mandate of the Declaration of Astana and facing a rising burden of noncommunicable diseases, policy-makers are reorienting their primary care systems to proactively address the social determinants of noncommunicable diseases. We have identified several promising primary care approaches for measuring and mobilizing action on social determinants of noncommunicable diseases. The evidence presented could assist care providers and policy-makers in considering which domains of social determinants of health to measure, which methods to use for collecting and collating this data, and how and at what level primary care organizations are positioned to intervene on local social determinants of health. Future research should examine undocumented innovators in this field and aspire to more rigorous observational and experimental study designs examining the impact of social determinants of health assessment on interventions to address local social determinants of health and health disparities.

## References

[1] Declaration of Astana on Primary Health Care in the 21st Century. Copenhagen: WHO Regional Office for Europe; 2018. Available from: http://www.euro.who.int/en/health-topics/Health-systems/primary-health-care/news/news/2018/11/revitalizing-primary-health-care-for-the-21st-century [cited 2020 Apr 17].

[2] Primary health care: towards universal health coverage. In: Seventy-second session of the World Health Assembly. Geneva: World Health Organization; 2019. Available from: https://apps.who.int/gb/ebwha/pdf_files/WHA72/A72_12-en.pdf [cited 2020 Apr 17].

[3] A/RES/74/2. Political declaration of the high-level meeting on universal health coverage. In: Seventy-fourth General Assembly. New York: United Nations; 2019. Available from: https://undocs.org/en/A/RES/74/2 [cited 2020 Apr 17].

[4] Binagwaho A, Adamo Ghebreyesus T. Primary healthcare is cornerstone of universal. BMJ. 2019 6 3;365:l2391. 10.1136/bmj.l2391 31160322

[5] Bishai DM, Cohen R, Alfonso YN, Adam T, Kuruvilla S, Schweitzer J. Factors contributing to maternal and child mortality reductions in 146 low- and middle-income countries between 1990 and 2010. PLoS One. 2016 1 19;11(1):e0144908. 10.1371/journal.pone.0144908 26783759 PMC4718632

[6] Hood CM, Gennuso KP, Swain GR, Catlin BB. County health rankings: relationships between determinant factors and health outcomes. Am J Prev Med. 2016 2;50(2):129–35. 26526164 10.1016/j.amepre.2015.08.024

[7] DeVoe JE, Bazemore AW, Cottrell EK, Likumahuwa-Ackman S, Grandmont J, Spach N, et al. Perspectives in primary care: a conceptual framework and path for integrating social determinants of health into primary care practice. Ann Fam Med. 2016 3;14(2):104–8. 10.1370/afm.1903 26951584 PMC4781512

[8] Allen L. Leveraging primary care to address social determinants. Lancet Public Health. 2018 10;3(10):e466. 10.1016/S2468-2667(18)30186-5 30314589

[9] Allen LN, Barry E, Gilbert C, Honney R, Turner-Moss E. How to move from managing sick individuals to creating healthy communities. Br J Gen Pract. 2019 1;69(678):8–9. 10.3399/bjgp19X700337 30591595 PMC6301354

[10] Primary health care: closing the gap between public health and primary care through integration. Geneva: World Health Organization; 2018. Available from: https://www.who.int/publications-detail/primary-health-care-closing-the-gap-between-public-health-and-primary-care-through-integration [cited 2020 Apr 17].

[11] Ensuring collaboration between primary health care and public health services. Copenhagen: World Health Organization Regional Office for Europe; 2018. Available from: http://www.euro.who.int/__data/assets/pdf_file/0009/389844/Designed-report-2.pdf?ua=1 [cited 2020 Apr 17].

[12] Towards a health promoting primary health care model in the WHO European Region (integrating primary health care and public health services). Intercountry retreat, 6–28 February 2020, Bled, Slovenia. Copenhagen: World Health Organization Regional Office for Europe; 2020. Available from: http://www.euro.who.int/en/media-centre/events/events/2020/02/intercountry-retreat-towards-a-health-promoting-primary-health-care-model-in-the-who-european-region-integrating-primary-health-care-and-public-health-services [cited 2020 Apr 17].

[13] Vos T, Abajobir AA, Abate KH, Abbafati C, Abbas KM, Abd-Allah F, et al.; GBD 2016 Disease and Injury Incidence and Prevalence Collaborators. Global, regional, and national incidence, prevalence, and years lived with disability for 328 diseases and injuries for 195 countries, 1990–2016: a systematic analysis for the Global Burden of Disease Study 2016. Lancet. 2017 9 16;390(10100):1211–59. 10.1016/S0140-6736(17)32154-2 28919117 PMC5605509

[14] Global Health Observatory (GHO) data: deaths from NCDs [internet]. Geneva: World Health Organization; 2020. Available from: https://www.who.int/gho/ncd/mortality_morbidity/ncd_total/en/ [cited 2020 Apr 17].

[15] Allen L, Williams J, Townsend N, Mikkelsen B, Roberts N, Foster C, et al. Socioeconomic status and non-communicable disease behavioural risk factors in low-income and lower-middle-income countries: a systematic review. Lancet Glob Health. 2017 3;5(3):e277–89. 10.1016/S2214-109X(17)30058-X 28193397 PMC5673683

[16] Williams J, Allen L, Wickramasinghe K, Mikkelsen B, Roberts N, Townsend N. A systematic review of associations between non-communicable diseases and socioeconomic status within low- and lower-middle-income countries. J Glob Health. 2018 12;8(2):020409. 10.7189/jogh.08.020409 30140435 PMC6076564

[17] Gilson L, Doherty J, Loewenson R, Francis V; WHO Commission on the Social Determinants of Health. Challenging inequity through health systems. London: Health Systems Knowledge Network; 2007 Available from: https://www.who.int/social_determinants/resources/csdh_media/hskn_final_2007_en.pdf [cited 2020 Apr 17].

[18] Closing the gap in a generation: health equity through action on the social determinants of health. Final report of the Commission on Social Determinants of Health. Geneva: World Health Organization; 2008. Available from: https://www.who.int/social_determinants/final_report/csdh_finalreport_2008.pdf [cited 2020 Apr 17].

[19] McGinnis JM, Williams-Russo P, Knickman JR. The case for more active policy attention to health promotion. Health Aff (Millwood). 2002 Mar-Apr;21(2):78–93. 10.1377/hlthaff.21.2.78 11900188

[20] Kuznetsova D. Healthy places: councils leading on public health. London: New Local Government Network; 2012 Available from: http://www.nlgn.org.uk/public/wp-content/uploads/Healthy-Places_FINAL.pdf [cited 2020 Apr 17].

[21] Bunker J, Frazier H, Mosteller F. The role of medical care in determining health: Creating an inventory of benefits. In: Amick I, editor. Society and health. Oxford: Oxford University Press; 1995 pp. 305–41.

[22] Dahlgren G, Whitehead M. European strategies for tackling social inequities in health. Levelling up part 2. Copenhagen: World Health Organization Regional Office for Europe; 2006 Available from: http://www.euro.who.int/__data/assets/pdf_file/0018/103824/E89384.pdf [cited 2020 Apr 17].

[23] Solar O, Irwin A. A conceptual framework for action on the social determinants of health: discussion paper for the Commission on Social Determinants of Health. Geneva: World Health Organization; 2010 Available from: https://www.who.int/sdhconference/resources/ConceptualframeworkforactiononSDH_eng.pdf [cited 2020 Apr 17].

[24] Integrating social care into the delivery of health care: moving upstream to improve the nation’s health. Washington: National Academies Press; 2019. 31940159

[25] Capturing social and behavioral domains and measures in electronic health records: phase 2. Washington: National Academies Press; 2015. Available from: https://www.ncbi.nlm.nih.gov/books/NBK268995/ [cited 2020 Apr 17]. 25590118

[26] Screening tools comparison [internet]. San Francisco: Social Intervention Research and Evaluation Network; 2019. Available from: https://sirenetwork.ucsf.edu/tools-resources/mmi/screening-tools-comparison [cited 2020 Apr 17].

[27] Arons A, DeSilvey S, Fichtenberg C, Gottlieb L. Documenting social determinants of health-related clinical activities using standardized medical vocabularies. JAMIA Open. 2018 12 24;2(1):81–8. 10.1093/jamiaopen/ooy051 31984347 PMC6951949

[28] Higgins JPT, Green S. Cochrane handbook for systematic reviews of interventions. Version 5.1.0. [updated March 2011]. London: Cochrane Collaboration; 2011 Available from: https://handbook-5-1.cochrane.org/ [cited 2020 Apr 17].

[29] Liberati A, Altman DG, Tetzlaff J, Mulrow C, Gøtzsche PC, Ioannidis JP, et al. The PRISMA statement for reporting systematic reviews and meta-analyses of studies that evaluate healthcare interventions: explanation and elaboration. BMJ. 2009 7 21;339 jul21 1:b2700. 10.1136/bmj.b2700 19622552 PMC2714672

[30] Allen LN, Smith RW, Simmons-Jones F, Roberts N, Honneye R, Currie J. Supplementary webappendix: Assessing and addressing the social determinants of noncommunicable diseases in primary care: a systematic review - Supplementary web appendix [data repository]. London: figshare; 2020. 10.6084/m9.figshare.12179085.v3 10.6084/m9.figshare.12179085.v3 PMC7607469 33177772

[31] Landis JR, Koch GG. The measurement of observer agreement for categorical data. Biometrics. 1977 3;33(1):159–74. 10.2307/2529310 843571

[32] Data collection form. London: Cochrane Effective Practice and Organisation of Care; 2017. Available from: https://epoc.cochrane.org/resources/epoc-resources-review-authors [cited 2019 Sep 3].

[33] Rose G. Sick individuals and sick populations. Int J Epidemiol. 1985 3;14(1):32–8. 10.1093/ije/14.1.32 3872850

[34] Green K, Zook M. When talking about social determinants, precision matters. Washington: Health Care Transformation Task Force; 2019 Available from: https:/hcttf.org/lets-get-it-right-sdoh/ [cited 2020 Apr 17].

[35] Alderwick H, Gottlieb LM. Meanings and misunderstandings: a social determinants of health lexicon for health care systems. Milbank Q. 2019 6;97(2):407–19. 10.1111/1468-0009.12390 31069864 PMC6554506

[36] Sterne JA, Hernán MA, Reeves BC, Savović J, Berkman ND, Viswanathan M, et al. ROBINS-I: a tool for assessing risk of bias in non-randomised studies of interventions. BMJ. 2016 10 12;355:i4919. 10.1136/bmj.i4919 27733354 PMC5062054

[37] Institute of Medicine. The Checkerboard area health system. In: Nutting PA, Connor EM, editors. Community oriented primary care: a practical assessment. Volume 2: Case studies Washington, DC: National Academies Press; 1984.

[38] Institute of Medicine. Crow Hill family medicine centre. In: Nutting PA, Connor EM, editors. Community oriented primary care: a practical assessment. Volume 2: Case studies Washington, DC: National Academies Press; 1984.

[39] Institute of Medicine. East Boston neighbourhood health centre. In: Nutting PA, Connor EM, editors. Community oriented primary care: a practical assessment. Volume 2. Case studies Washington, DC: National Academies Press; 1984.

[40] Institute of Medicine. Montefiore family health center. In: Nutting PA, Connor EM, editors. Community oriented primary care: a practical assessment. Volume 2: Case studies Washington, DC: National Academies Press; 1984.

[41] Institute of Medicine. Tarboro-Edgecombe health services system. In: Nutting PA, Connor EM, editors. Community oriented primary care: a practical assessment. Volume 2: Case studies Washington, DC: National Academies Press; 1984.

[42] Tollman SM. The Pholela health centre – the origins of community-oriented primary health care (COPC). An appreciation of the work of Sidney and Emily Kark. South African Med. S Afr Med J. 1994 10;84(10):653–8. 7839251

[43] Williams RL, Jaén CR. Tools for community-oriented primary care: use of key informant trees in eleven practices. J Natl Med Assoc. 2000 4;92(4):157–62. 10976171 PMC2640606

[44] Fone D, Jones A, Watkins J, Lester N, Cole J, Thomas G, et al. Using local authority data for action on health inequalities: the Caerphilly Health and Social Needs Study. Br J Gen Pract. 2002 10;52(483):799–804. 12392118 PMC1316081

[45] Horne M, Costello J. A public health approach to health needs assessment at the interface of primary care and community development : findings from an action research study. Prim Health Care Res Dev. 2003;4(4):340–52. 10.1191/1463423603pc173oa

[46] Bam N, Marcus T, Hugo J, Kinkel HF. Conceptualizing community oriented primary care (COPC) – the Tshwane, South Africa, health post model. Afr J Prim Health Care Fam Med. 2013;5(1):54–6. 10.4102/phcfm.v5i1.423

[47] Hardt NS, Muhamed S, Das R, Estrella R, Roth J. Neighborhood-level hot spot maps to inform delivery of primary care and allocation of social resources. Perm J. 2013 Winter;17(1):4–9. 10.7812/TPP/12-090 23596361 PMC3627788

[48] Gottlieb LM, Tirozzi KJ, Manchanda R, Burns AR, Sandel MT. Moving electronic medical records upstream: incorporating social determinants of health. Am J Prev Med. 2015 2;48(2):215–8. 10.1016/j.amepre.2014.07.009 25217095

[49] Jinabhai CC, Marcus TS, Chaponda A. Rapid appraisal of ward based outreach teams. Pretoria: Albertina Sisulu Executive Leadership Programme in Health; 2015 Available from: https://www.up.ac.za/media/shared/62/COPC/COPC%20Reports%20Publications/wbot-report-epub-lr-2.zp86437.pdf [cited 2020 Apr 17].

[50] Page-Reeves J, Kaufman W, Bleecker M, Norris J, McCalmont K, Ianakieva V, et al. Addressing social determinants of health in a clinic setting: the WellRx pilot in Albuquerque, New Mexico. J Am Board Fam Med. 2016 May-Jun;29(3):414–8. 10.3122/jabfm.2016.03.150272 27170801

[51] Pinto AD, Glattstein-Young G, Mohamed A, Bloch G, Leung FH, Glazier RH. Building a foundation to reduce health inequities: routine collection of sociodemographic data in primary care. J Am Board Fam Med. 2016 May-Jun;29(3):348–55. 10.3122/jabfm.2016.03.150280 27170792

[52] Lofters AK, Schuler A, Slater M, Baxter NN, Persaud N, Pinto AD, et al. Using self-reported data on the social determinants of health in primary care to identify cancer screening disparities: opportunities and challenges. BMC Fam Pract. 2017 2 28;18(1):31. 10.1186/s12875-017-0599-z 28241787 PMC5330155

[53] Pinto AD, Bloch G. Framework for building primary care capacity to address the social determinants of health. Can Fam Physician. 2017 11;63(11):e476–82. 29138172 PMC5685463

[54] Andermann A. Screening for social determinants of health in clinical care: moving from the margins to the mainstream. Public Health Rev. 2018 6 22;39(1):19. 10.1186/s40985-018-0094-7 29977645 PMC6014006

[55] Sokol R, Austin A, Chandler C, Byrum E, Bousquette J, Lancaster C, et al. Screening children for social determinants of health: a systematic review. Pediatrics. 2019 10;144(4):e20191622. 10.1542/peds.2019-1622 31548335 PMC6996928

[56] Ellis DA, McQueenie R, McConnachie A, Wilson P, Williamson AE. Demographic and practice factors predicting repeated non-attendance in primary care: a national retrospective cohort analysis. Lancet Public Health. 2017 12;2(12):e551–9. 10.1016/S2468-2667(17)30217-7 29253440 PMC5725414

[57] Williamson AE, Ellis DA, Wilson P, McQueenie R, McConnachie A. Understanding repeated non-attendance in health services: a pilot analysis of administrative data and full study protocol for a national retrospective cohort. BMJ Open. 2017 2 14;7(2):e014120. 10.1136/bmjopen-2016-014120 28196951 PMC5319001

[58] Garg A, Boynton-Jarrett R, Dworkin PH. Avoiding the unintended consequences of screening for social determinants of health. JAMA. 2016 8 23-30;316(8):813–4. 10.1001/jama.2016.9282 27367226

[59] Gottlieb LM, Francis DE, Beck AF. Uses and misuses of patient- and neighborhood-level social determinants of health data. Perm J. 2018;22:18–078. 30227912 10.7812/TPP/18-078 PMC6141653

[60] A vision for primary care in the 21st century. Geneva: World Health Organization; 2019. Available from: https://apps.who.int/iris/bitstream/handle/10665/328065/WHO-HIS-SDS-2018.15-eng.pdf [cited 2020 Apr 17].

[61] Fingertips data: national general practice profiles [internet]. London: Public Health England; 2020. Available from: https://fingertips.phe.org.uk/profile/general-practice [cited 2020 Apr 17].

[62] PHATE. The Population Health Assessment Engine [internet]. Lexington: American Board of Family Medicine; 2019. Available from: https://primeregistry.org/phate/ [cited 2020 Apr 17].

[63] Health in the municipality. Ljubljana: Slovenian National Institute of Public Health; 2020. Slovenian. Available from: http://obcine.nijz.si/Default.aspx?leto=2019&lang=ang [cited 2020 Apr 17].

[64] Elias RR, Jutte DP, Moore A. Exploring consensus across sectors for measuring the social determinants of health. SSM Popul Health. 2019 4 9;7:100395. 10.1016/j.ssmph.2019.100395 31049390 PMC6484213

[65] WHO Commission on the Social Determinants of Health. A conceptual framework for action on the social determinants of health. Geneva: World Health Organization; 2007. Available from: https://www.who.int/sdhconference/resources/ConceptualframeworkforactiononSDH_eng.pdf [cited 2020 Apr 17].

[66] What are the advantages and disadvantages of restructuring a health care system to be more focused on primary care services? Copenhagen: World Health Organization Regional Office for Europe; 2004. Available from: http://www.euro.who.int/__data/assets/pdf_file/0004/74704/E82997.pdf?ua=1 [cited 2020 Apr 17].

[67] van Wijck F. [The first line after the Woudschoten.] Rotterdam: DeEerstelijns; 2019. Dutch. Available from: https://www.de-eerstelijns.nl/wp-content/uploads/2019/03/DEL-nr2_2019_Woudschoten.pdf [cited 2020 Apr 17].

[68] Kaffash J. PCNs have been received “incredibly well”, says Hancock [internet]. Pulse. 2019 Nov 18. Available from: http://www.pulsetoday.co.uk/news/pcns-have-been-received-incredibly-well-says-hancock/20039714.article [cited 2020 Apr 17].

[69] Network Contract Directed Enhanced Service (DES) guidance 2019/20. London: NHS England; 2019. Available from: https://www.england.nhs.uk/publication/network-contract-directed-enhanced-service-des-guidance-2019-20/ [cited 2020 Apr 17].

[70] Bambra C, Gibson M, Sowden A, Wright K, Whitehead M, Petticrew M. Tackling the wider social determinants of health and health inequalities: evidence from systematic reviews. J Epidemiol Community Health. 2010 4;64(4):284–91. 10.1136/jech.2008.082743 19692738 PMC2921286

[71] Rutter H, Savona N, Glonti K, Bibby J, Cummins S, Finegood DT, et al. The need for a complex systems model of evidence for public health. Lancet. 2017 12 9;390(10112):2602–4. 10.1016/S0140-6736(17)31267-9 28622953

[72] Allen LN, Barkley S, De Maeseneer J, van Weel C, Kluge H, de Wit N, et al. Unfulfilled potential of primary care in Europe. BMJ. 2018 10 24;363:k4469. 10.1136/bmj.k4469 30355571

[73] Integrating social care into the delivery of health care: moving upstream to improve the nation’s health. Washington: National Academies of Sciences Medicine and Engineering; 2019. Available from: https://www.nap.edu/catalog/25467/integrating-social-care-into-the-delivery-of-health-care-moving [cited 2020 Apr 17]. 31940159

[74] Gottlieb LM, Wing H, Adler NE. A systematic review of interventions on patients’ social and economic needs. Am J Prev Med. 2017 11;53(5):719–29. 10.1016/j.amepre.2017.05.011 28688725

